# 4-[3-(Phen­oxy­meth­yl)-7*H*-1,2,4-triazolo[3,4-*b*][1,3,4]thia­diazin-6-yl]-3-(*p*-tol­yl)sydnone

**DOI:** 10.1107/S1600536810029910

**Published:** 2010-07-31

**Authors:** Jia Hao Goh, Hoong-Kun Fun, Nithinchandra, B. Kalluraya

**Affiliations:** a X-ray Crystallography Unit, School of Physics, Universiti Sains Malaysia, 11800 USM, Penang, Malaysia; b Department of Studies in Chemistry, Mangalore University, Mangalagangotri, Mangalore 574 199, India

## Abstract

In the title triazolothia­diazine derivative, C_20_H_16_N_6_O_3_S {systematic name: 3-(4-methyl­phen­yl)-4-[3-(phen­oxy­meth­yl)-7*H*-1,2,4-triazolo[3,4-*b*][1,3,4]thia­diazin-6-yl]-1,2,3-oxadiazol-3-ium-5-olate}, an *S*(6) ring motif is generated by an intra­molecular C—H⋯O hydrogen bond. The 3,6-dihydro-1,3,4-thia­diazine ring adopts a twist-boat conformation. The dihedral angle between the 1,2,3-oxadiazole and 1,2,4-triazole rings is 46.45 (14)°. The 1,2,3-oxadiazole ring is inclined at dihedral angle of 59.49 (13)° with respect to the benzene ring attached to it. In the crystal structure, inter­molecular C—H⋯O and C—H⋯N hydrogen bonds link neighbouring mol­ecules into two-mol­ecule-thick arrays parallel to the *bc* plane. A short S⋯O inter­action [2.9565 (19) Å] also occurs.

## Related literature

For general background to and applications of materials related to the title compound, see: Kalluraya & Rahiman (1997 ▶); Newton & Ramsden (1982 ▶); Wagner & Hill (1974 ▶). For graph-set descriptions of hydrogen-bond ring motifs, see: Bernstein *et al.* (1995 ▶). For closely related structures, see: Goh *et al.* (2010**a* ▶,*b* ▶,c* ▶). For the stability of the temperature controller used in the data collection, see: Cosier & Glazer (1986 ▶). For puckering parameters, see: Cremer & Pople (1975 ▶).
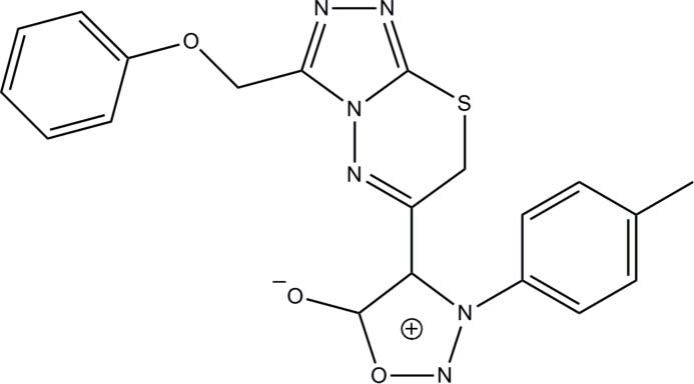


## Experimental

### 

#### Crystal data

C_20_H_16_N_6_O_3_S
*M* *_r_* = 420.45
Monoclinic, 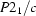

*a* = 20.6555 (7) Å
*b* = 8.1918 (3) Å
*c* = 11.1979 (4) Å
β = 96.127 (2)°
*V* = 1883.93 (12) Å^3^
*Z* = 4
Mo *K*α radiation
μ = 0.21 mm^−1^
*T* = 100 K
0.26 × 0.13 × 0.07 mm

#### Data collection

Bruker SMART APEXII CCD diffractometer
Absorption correction: multi-scan (*SADABS*; Bruker, 2009 ▶) *T*_min_ = 0.947, *T*_max_ = 0.985
17653 measured reflections
4318 independent reflections
2829 reflections with *I* > 2σ(*I*)
*R*_int_ = 0.065

#### Refinement

*R*[*F* ^2^ > 2σ(*F* ^2^)] = 0.060
*wR*(*F* ^2^) = 0.133
*S* = 1.03
4318 reflections
272 parameters
H-atom parameters constrained
Δρ_max_ = 0.67 e Å^−3^
Δρ_min_ = −0.53 e Å^−3^

### 

Data collection: *APEX2* (Bruker, 2009 ▶); cell refinement: *SAINT* (Bruker, 2009 ▶); data reduction: *SAINT*; program(s) used to solve structure: *SHELXTL* (Sheldrick, 2008 ▶); program(s) used to refine structure: *SHELXTL*; molecular graphics: *SHELXTL*; software used to prepare material for publication: *SHELXTL* and *PLATON* (Spek, 2009 ▶).

## Supplementary Material

Crystal structure: contains datablocks global, I. DOI: 10.1107/S1600536810029910/hb5566sup1.cif

Structure factors: contains datablocks I. DOI: 10.1107/S1600536810029910/hb5566Isup2.hkl

Additional supplementary materials:  crystallographic information; 3D view; checkCIF report

## Figures and Tables

**Table 1:** Hydrogen-bond geometry (Å, °)

*D*—H⋯*A*	*D*—H	H⋯*A*	*D*⋯*A*	*D*—H⋯*A*
C10—H10*A*⋯O3	0.97	2.26	3.026 (3)	135
C10—H10*A*⋯O3^i^	0.97	2.55	3.165 (3)	122
C10—H10*B*⋯O3^ii^	0.97	2.44	3.279 (3)	145
C19—H19*A*⋯N5^iii^	0.93	2.61	3.491 (3)	158

Symmetry codes: (i) 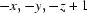; (ii) 
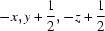
; (iii) 
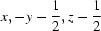
.

**Fig. 1.**
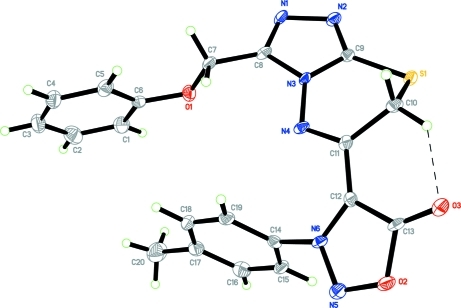
The molecular structure of (I), showing 50% probability displacement ellipsoids for non-H atoms. An intramolecular hydrogen bond is shown as dashed line.

**Fig. 2.**
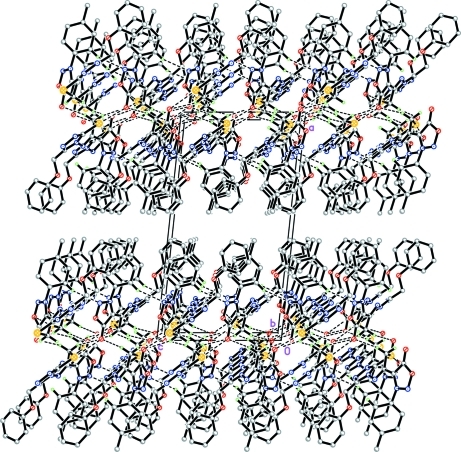
The crystal structure of (I), viewed along the b axis, showing two-molecule-thick arrays parallel to the bc plane. Hydrogen atoms not involved in intermolecular interactions (dashed lines) have been omitted for clarity.

## supplementary crystallographic information

### Comment

Sydnones are a novel class of mesoionic compounds consisting of 1,2,3-oxadiazole ring system. A number of sydnone derivatives have shown diverse biological activities such as anti-inflammatory, analgesic and anti-arthritic (Newton & Ramsden, 1982; Wagner & Hill, 1974) properties. Sydnones possessing heterocyclic moieties at the 4-position are also known for a wide range of biological properties (Kalluraya & Rahiman, 1997). Encouraged by these reports and in continuation of our research for biologically active nitrogen containing heterocycles, a triazolothiadiazine moiety at the 4-position of the phenylsydnone was introduced.

In the title triazolothiadiazine derivative, an intramolecular C10—H10A···O3 hydrogen bond (Table 1) generates a six-membered ring, producing an *S*(6) hydrogen bond ring motif (Fig. 1, Bernstein *et al.*, 1995). The 3,6-dihydro-1,3,4-thiadiazine ring (C9-C11/N3/N4/S1) adopts twist-boat conformation, with puckering parameters of Q = 0.634 (2) Å, θ = 67.08 (18)° and φ = 322.1 (2)° (Cremer & Pople, 1975). The dihedral angle formed between these essentially planar 1,2,3-oxadiazole (C12/C13/O2/N5/N6) and 1,2,4-triazole (C8/N1/N2/C9/N3) rings is 46.45 (14)°. The C1-C6 and C14-C19 phenyl rings are inclined at dihedral angles of 77.56 (14) and 59.49 (13)°, respectively, with respect to 1,2,3-oxadiazole and 1,2,4-triazole rings. The geometric parameters are consistent to those observed in closely related structures (Goh *et al.*, 2010**a*,*b*,c*).

In the crystal structure, intermolecular C10—H10A···O3, C10—H10B···O3 and C19—H19A···N5 hydrogen bonds (Table 1) interconnect neighbouring molecules into two-molecule-thick arrays parallel to the *bc* plane (Fig. 2). The interesting feature of the crystal structure is the intermolecular short S1···O3 interaction [2.9565 (19) Å; symmetry code: -x, -y, -z+1], which is significantly shorter than the sum of Van der Waals radii of the relevant atoms, further stabilizing the crystal structure.

### Experimental

A solution of triazole (0.01 mol) and 4-bromoacetyl-3-tolylsydnone (0.01 mol) in absolute ethanol (20 ml) was heated under reflux for 10–12 h. The solution was concentrated, cooled to room temperature and neutrallized with 10 % sodium bicarbonate solution. The solid separated was filtered, washed with water, dried and recrystallized from ethanol. Colourless blocks of (I) were obtained from a 1:2 mixture of DMF and ethanol by slow evaporation.

### Refinement

All hydrogen atoms were placed in their calculated positions, with C—H = 0.93–0.97 Å, and refined using a riding model, with *U*_iso_ = 1.2 or 1.5 *U*_eq_(C). The rotating group model was used for the methyl group.

### Crystal data

**Table:**

C_20_H_16_N_6_O_3_S	*F*(000) = 872
*M**_r_* = 420.45	*D*_x_ = 1.482 Mg m^−^^3^
Monoclinic, *P*2_1_/*c*	Mo *K*α radiation, λ = 0.71073 Å
Hall symbol: -P 2ybc	Cell parameters from 4257 reflections
*a* = 20.6555 (7) Å	θ = 2.7–30.6°
*b* = 8.1918 (3) Å	µ = 0.21 mm^−^^1^
*c* = 11.1979 (4) Å	*T* = 100 K
β = 96.127 (2)°	Block, colourless
*V* = 1883.93 (12) Å^3^	0.26 × 0.13 × 0.07 mm
*Z* = 4	

### Data collection

**Table:**

Bruker SMART APEXII CCD diffractometer	4318 independent reflections
Radiation source: fine-focus sealed tube	2829 reflections with *I* > 2σ(*I*)
graphite	*R*_int_ = 0.065
φ and ω scans	θ_max_ = 27.5°, θ_min_ = 2.0°
Absorption correction: multi-scan (*SADABS*; Bruker, 2009)	*h* = −26→26
*T*_min_ = 0.947, *T*_max_ = 0.985	*k* = −10→10
17653 measured reflections	*l* = −14→14

### Refinement

**Table:**

Refinement on *F*^2^	Primary atom site location: structure-invariant direct methods
Least-squares matrix: full	Secondary atom site location: difference Fourier map
*R*[*F*^2^ > 2σ(*F*^2^)] = 0.060	Hydrogen site location: inferred from neighbouring sites
*wR*(*F*^2^) = 0.133	H-atom parameters constrained
*S* = 1.03	*w* = 1/[σ^2^(*F*_o_^2^) + (0.060*P*)^2^ + 0.4167*P*] where *P* = (*F*_o_^2^ + 2*F*_c_^2^)/3
4318 reflections	(Δ/σ)_max_ = 0.003
272 parameters	Δρ_max_ = 0.67 e Å^−^^3^
0 restraints	Δρ_min_ = −0.53 e Å^−^^3^

### Special details

**Table:**

Experimental. The crystal was placed in the cold stream of an Oxford Cryosystems Cobra open-flow nitrogen cryostat (Cosier & Glazer, 1986) operating at 100.0 (1)K.
Geometry. All esds (except the esd in the dihedral angle between two l.s. planes) are estimated using the full covariance matrix. The cell esds are taken into account individually in the estimation of esds in distances, angles and torsion angles; correlations between esds in cell parameters are only used when they are defined by crystal symmetry. An approximate (isotropic) treatment of cell esds is used for estimating esds involving l.s. planes.
Refinement. Refinement of F^2^ against ALL reflections. The weighted R-factor wR and goodness of fit S are based on F^2^, conventional R-factors R are based on F, with F set to zero for negative F^2^. The threshold expression of F^2^ > 2sigma(F^2^) is used only for calculating R-factors(gt) etc. and is not relevant to the choice of reflections for refinement. R-factors based on F^2^ are statistically about twice as large as those based on F, and R- factors based on ALL data will be even larger.

### Fractional atomic coordinates and isotropic or equivalent isotropic displacement parameters (Å^2^)

**Table:**

	*x*	*y*	*z*	*U*_iso_*/*U*_eq_	
S1	0.06014 (3)	0.33229 (8)	0.37690 (6)	0.01451 (18)	
O1	0.28045 (8)	0.2544 (2)	0.08679 (15)	0.0183 (5)	
O2	0.10735 (9)	−0.3314 (2)	0.48621 (15)	0.0174 (4)	
O3	0.01271 (9)	−0.1961 (2)	0.43267 (15)	0.0168 (4)	
N1	0.16326 (10)	0.4933 (3)	0.13016 (18)	0.0159 (5)	
N2	0.12096 (10)	0.5222 (3)	0.21876 (19)	0.0159 (5)	
N3	0.14032 (10)	0.2588 (3)	0.20962 (18)	0.0119 (5)	
N4	0.15133 (10)	0.0982 (3)	0.24765 (18)	0.0134 (5)	
N5	0.17122 (11)	−0.3211 (3)	0.46629 (19)	0.0167 (5)	
N6	0.17491 (10)	−0.1933 (3)	0.39705 (18)	0.0130 (5)	
C1	0.38786 (14)	0.2084 (4)	0.0490 (2)	0.0226 (7)	
H1A	0.4012	0.2624	0.1204	0.027*	
C2	0.43384 (14)	0.1464 (4)	−0.0207 (3)	0.0282 (8)	
H2A	0.4779	0.1602	0.0040	0.034*	
C3	0.41449 (14)	0.0641 (4)	−0.1269 (3)	0.0263 (7)	
H3A	0.4453	0.0212	−0.1730	0.032*	
C4	0.34920 (14)	0.0469 (4)	−0.1629 (3)	0.0235 (7)	
H4A	0.3361	−0.0070	−0.2345	0.028*	
C5	0.30230 (14)	0.1081 (4)	−0.0949 (2)	0.0194 (7)	
H5A	0.2583	0.0949	−0.1202	0.023*	
C6	0.32231 (13)	0.1897 (3)	0.0121 (2)	0.0168 (6)	
C7	0.21295 (12)	0.2477 (3)	0.0432 (2)	0.0149 (6)	
H7A	0.1988	0.1348	0.0363	0.018*	
H7B	0.2060	0.2974	−0.0357	0.018*	
C8	0.17459 (12)	0.3361 (3)	0.1279 (2)	0.0142 (6)	
C9	0.10811 (12)	0.3793 (3)	0.2631 (2)	0.0123 (6)	
C10	0.04330 (12)	0.1268 (3)	0.3200 (2)	0.0148 (6)	
H10A	0.0185	0.0674	0.3746	0.018*	
H10B	0.0175	0.1323	0.2424	0.018*	
C11	0.10610 (12)	0.0393 (3)	0.3078 (2)	0.0125 (6)	
C12	0.11699 (12)	−0.1155 (3)	0.3677 (2)	0.0123 (6)	
C13	0.07074 (14)	−0.2057 (3)	0.4260 (2)	0.0143 (6)	
C14	0.24014 (12)	−0.1537 (3)	0.3708 (2)	0.0137 (6)	
C15	0.28634 (13)	−0.1262 (3)	0.4668 (2)	0.0182 (6)	
H15A	0.2749	−0.1266	0.5449	0.022*	
C16	0.35008 (14)	−0.0980 (4)	0.4450 (3)	0.0228 (7)	
H16A	0.3817	−0.0798	0.5092	0.027*	
C17	0.36760 (14)	−0.0964 (4)	0.3281 (3)	0.0213 (7)	
C18	0.31939 (13)	−0.1232 (3)	0.2334 (2)	0.0206 (7)	
H18A	0.3305	−0.1209	0.1551	0.025*	
C19	0.25567 (13)	−0.1529 (3)	0.2528 (2)	0.0178 (6)	
H19A	0.2240	−0.1718	0.1889	0.021*	
C20	0.43702 (14)	−0.0674 (4)	0.3043 (3)	0.0351 (8)	
H20D	0.4422	−0.0966	0.2229	0.053*	
H20A	0.4655	−0.1330	0.3581	0.053*	
H20B	0.4476	0.0458	0.3169	0.053*	

### Atomic displacement parameters (Å^2^)

**Table:**

	*U*^11^	*U*^22^	*U*^33^	*U*^12^	*U*^13^	*U*^23^
S1	0.0213 (4)	0.0098 (4)	0.0135 (3)	0.0003 (3)	0.0064 (2)	0.0003 (3)
O1	0.0180 (10)	0.0225 (12)	0.0146 (9)	0.0027 (9)	0.0028 (7)	−0.0026 (9)
O2	0.0265 (11)	0.0118 (11)	0.0144 (9)	0.0002 (9)	0.0041 (8)	0.0034 (9)
O3	0.0196 (10)	0.0162 (11)	0.0150 (9)	−0.0027 (9)	0.0040 (7)	0.0016 (9)
N1	0.0233 (13)	0.0158 (14)	0.0094 (10)	−0.0014 (11)	0.0052 (9)	0.0023 (11)
N2	0.0229 (13)	0.0122 (13)	0.0130 (11)	0.0001 (11)	0.0039 (9)	0.0019 (11)
N3	0.0174 (12)	0.0089 (12)	0.0105 (10)	−0.0002 (10)	0.0059 (9)	0.0032 (10)
N4	0.0242 (13)	0.0075 (12)	0.0084 (10)	0.0003 (10)	0.0017 (9)	0.0002 (10)
N5	0.0247 (13)	0.0109 (13)	0.0148 (11)	−0.0014 (11)	0.0039 (9)	−0.0016 (11)
N6	0.0238 (12)	0.0072 (12)	0.0079 (10)	−0.0003 (10)	0.0015 (9)	−0.0003 (10)
C1	0.0280 (16)	0.0200 (17)	0.0193 (14)	0.0012 (14)	0.0003 (12)	−0.0037 (14)
C2	0.0205 (16)	0.030 (2)	0.0342 (17)	0.0029 (14)	0.0025 (13)	0.0007 (17)
C3	0.0305 (18)	0.0229 (18)	0.0278 (16)	0.0045 (15)	0.0143 (13)	0.0008 (15)
C4	0.0322 (17)	0.0199 (17)	0.0193 (14)	0.0023 (15)	0.0079 (12)	−0.0012 (14)
C5	0.0237 (15)	0.0181 (17)	0.0169 (14)	−0.0030 (13)	0.0040 (11)	0.0005 (14)
C6	0.0217 (15)	0.0133 (16)	0.0161 (13)	0.0035 (13)	0.0058 (11)	0.0055 (13)
C7	0.0207 (14)	0.0135 (16)	0.0109 (12)	0.0000 (12)	0.0039 (10)	0.0030 (12)
C8	0.0195 (14)	0.0126 (15)	0.0107 (12)	−0.0032 (13)	0.0015 (10)	0.0031 (13)
C9	0.0167 (14)	0.0087 (15)	0.0117 (12)	−0.0004 (12)	0.0020 (10)	−0.0001 (12)
C10	0.0205 (14)	0.0086 (15)	0.0156 (13)	−0.0019 (12)	0.0025 (11)	0.0021 (12)
C11	0.0177 (14)	0.0122 (15)	0.0074 (12)	−0.0003 (12)	0.0009 (10)	−0.0039 (12)
C12	0.0164 (14)	0.0105 (15)	0.0103 (12)	0.0006 (12)	0.0028 (10)	−0.0036 (12)
C13	0.0304 (16)	0.0058 (15)	0.0070 (12)	0.0004 (13)	0.0035 (11)	−0.0005 (12)
C14	0.0172 (14)	0.0087 (15)	0.0154 (13)	0.0023 (12)	0.0023 (10)	−0.0015 (13)
C15	0.0287 (16)	0.0152 (16)	0.0111 (12)	0.0022 (13)	0.0040 (11)	0.0014 (13)
C16	0.0243 (16)	0.0198 (17)	0.0236 (15)	0.0013 (14)	−0.0002 (12)	−0.0005 (15)
C17	0.0239 (16)	0.0148 (16)	0.0265 (16)	0.0010 (13)	0.0083 (12)	0.0040 (14)
C18	0.0293 (16)	0.0177 (17)	0.0164 (13)	0.0048 (14)	0.0097 (12)	0.0033 (13)
C19	0.0291 (16)	0.0108 (16)	0.0134 (13)	0.0039 (13)	0.0023 (11)	0.0005 (13)
C20	0.0268 (17)	0.043 (2)	0.0368 (19)	−0.0018 (17)	0.0080 (14)	0.0098 (18)

### Geometric parameters (Å, °)

**Table:**

S1—C9	1.739 (3)	C4—H4A	0.9300
S1—C10	1.820 (3)	C5—C6	1.395 (4)
O1—C6	1.372 (3)	C5—H5A	0.9300
O1—C7	1.428 (3)	C7—C8	1.487 (4)
O2—N5	1.364 (3)	C7—H7A	0.9700
O2—C13	1.406 (3)	C7—H7B	0.9700
O3—C13	1.212 (3)	C10—C11	1.501 (4)
N1—C8	1.310 (3)	C10—H10A	0.9700
N1—N2	1.410 (3)	C10—H10B	0.9700
N2—C9	1.310 (3)	C11—C12	1.441 (4)
N3—C9	1.364 (3)	C12—C13	1.420 (4)
N3—C8	1.371 (3)	C14—C15	1.378 (4)
N3—N4	1.395 (3)	C14—C19	1.393 (3)
N4—C11	1.301 (3)	C15—C16	1.384 (4)
N5—N6	1.310 (3)	C15—H15A	0.9300
N6—C12	1.364 (3)	C16—C17	1.395 (4)
N6—C14	1.446 (3)	C16—H16A	0.9300
C1—C6	1.381 (4)	C17—C18	1.392 (4)
C1—C2	1.388 (4)	C17—C20	1.505 (4)
C1—H1A	0.9300	C18—C19	1.378 (4)
C2—C3	1.389 (4)	C18—H18A	0.9300
C2—H2A	0.9300	C19—H19A	0.9300
C3—C4	1.373 (4)	C20—H20D	0.9600
C3—H3A	0.9300	C20—H20A	0.9600
C4—C5	1.389 (4)	C20—H20B	0.9600
			
C9—S1—C10	92.89 (12)	N2—C9—S1	129.0 (2)
C6—O1—C7	115.64 (19)	N3—C9—S1	120.3 (2)
N5—O2—C13	110.89 (19)	C11—C10—S1	109.82 (18)
C8—N1—N2	107.7 (2)	C11—C10—H10A	109.7
C9—N2—N1	106.4 (2)	S1—C10—H10A	109.7
C9—N3—C8	105.7 (2)	C11—C10—H10B	109.7
C9—N3—N4	128.3 (2)	S1—C10—H10B	109.7
C8—N3—N4	124.1 (2)	H10A—C10—H10B	108.2
C11—N4—N3	113.9 (2)	N4—C11—C12	118.8 (2)
N6—N5—O2	105.33 (19)	N4—C11—C10	123.0 (2)
N5—N6—C12	114.3 (2)	C12—C11—C10	118.1 (2)
N5—N6—C14	114.4 (2)	N6—C12—C13	105.2 (2)
C12—N6—C14	131.2 (2)	N6—C12—C11	127.8 (2)
C6—C1—C2	120.0 (3)	C13—C12—C11	126.3 (2)
C6—C1—H1A	120.0	O3—C13—O2	120.2 (2)
C2—C1—H1A	120.0	O3—C13—C12	135.5 (3)
C1—C2—C3	120.5 (3)	O2—C13—C12	104.3 (2)
C1—C2—H2A	119.8	C15—C14—C19	121.9 (2)
C3—C2—H2A	119.8	C15—C14—N6	117.5 (2)
C4—C3—C2	119.0 (3)	C19—C14—N6	120.5 (2)
C4—C3—H3A	120.5	C14—C15—C16	118.8 (2)
C2—C3—H3A	120.5	C14—C15—H15A	120.6
C3—C4—C5	121.5 (3)	C16—C15—H15A	120.6
C3—C4—H4A	119.2	C15—C16—C17	120.9 (3)
C5—C4—H4A	119.2	C15—C16—H16A	119.5
C4—C5—C6	119.0 (3)	C17—C16—H16A	119.5
C4—C5—H5A	120.5	C18—C17—C16	118.5 (3)
C6—C5—H5A	120.5	C18—C17—C20	120.6 (3)
O1—C6—C1	115.9 (2)	C16—C17—C20	121.0 (3)
O1—C6—C5	124.1 (2)	C19—C18—C17	121.7 (2)
C1—C6—C5	120.0 (3)	C19—C18—H18A	119.1
O1—C7—C8	109.3 (2)	C17—C18—H18A	119.1
O1—C7—H7A	109.8	C18—C19—C14	118.1 (2)
C8—C7—H7A	109.8	C18—C19—H19A	121.0
O1—C7—H7B	109.8	C14—C19—H19A	121.0
C8—C7—H7B	109.8	C17—C20—H20D	109.5
H7A—C7—H7B	108.3	C17—C20—H20A	109.5
N1—C8—N3	109.6 (2)	H20D—C20—H20A	109.5
N1—C8—C7	126.9 (2)	C17—C20—H20B	109.5
N3—C8—C7	123.3 (2)	H20D—C20—H20B	109.5
N2—C9—N3	110.6 (2)	H20A—C20—H20B	109.5
			
C8—N1—N2—C9	−1.4 (3)	C9—S1—C10—C11	54.65 (19)
C9—N3—N4—C11	30.0 (3)	N3—N4—C11—C12	−170.4 (2)
C8—N3—N4—C11	−167.9 (2)	N3—N4—C11—C10	8.3 (3)
C13—O2—N5—N6	0.5 (2)	S1—C10—C11—N4	−53.7 (3)
O2—N5—N6—C12	−0.3 (3)	S1—C10—C11—C12	125.0 (2)
O2—N5—N6—C14	176.24 (18)	N5—N6—C12—C13	0.1 (3)
C6—C1—C2—C3	−0.6 (5)	C14—N6—C12—C13	−175.8 (2)
C1—C2—C3—C4	0.9 (5)	N5—N6—C12—C11	170.7 (2)
C2—C3—C4—C5	−0.8 (4)	C14—N6—C12—C11	−5.2 (4)
C3—C4—C5—C6	0.4 (4)	N4—C11—C12—N6	18.5 (4)
C7—O1—C6—C1	174.7 (2)	C10—C11—C12—N6	−160.3 (2)
C7—O1—C6—C5	−5.8 (4)	N4—C11—C12—C13	−172.8 (2)
C2—C1—C6—O1	179.8 (3)	C10—C11—C12—C13	8.4 (4)
C2—C1—C6—C5	0.2 (4)	N5—O2—C13—O3	179.4 (2)
C4—C5—C6—O1	−179.7 (3)	N5—O2—C13—C12	−0.4 (2)
C4—C5—C6—C1	−0.1 (4)	N6—C12—C13—O3	−179.6 (3)
C6—O1—C7—C8	−174.6 (2)	C11—C12—C13—O3	9.6 (5)
N2—N1—C8—N3	1.3 (3)	N6—C12—C13—O2	0.2 (3)
N2—N1—C8—C7	175.7 (2)	C11—C12—C13—O2	−170.6 (2)
C9—N3—C8—N1	−0.8 (3)	N5—N6—C14—C15	−56.8 (3)
N4—N3—C8—N1	−166.3 (2)	C12—N6—C14—C15	119.1 (3)
C9—N3—C8—C7	−175.4 (2)	N5—N6—C14—C19	119.8 (3)
N4—N3—C8—C7	19.1 (4)	C12—N6—C14—C19	−64.4 (4)
O1—C7—C8—N1	84.4 (3)	C19—C14—C15—C16	−0.4 (4)
O1—C7—C8—N3	−101.9 (3)	N6—C14—C15—C16	176.1 (2)
N1—N2—C9—N3	0.8 (3)	C14—C15—C16—C17	0.3 (4)
N1—N2—C9—S1	178.78 (18)	C15—C16—C17—C18	0.3 (4)
C8—N3—C9—N2	−0.1 (3)	C15—C16—C17—C20	−179.4 (3)
N4—N3—C9—N2	164.6 (2)	C16—C17—C18—C19	−0.9 (4)
C8—N3—C9—S1	−178.20 (18)	C20—C17—C18—C19	178.8 (3)
N4—N3—C9—S1	−13.6 (3)	C17—C18—C19—C14	0.8 (4)
C10—S1—C9—N2	154.7 (2)	C15—C14—C19—C18	−0.1 (4)
C10—S1—C9—N3	−27.6 (2)	N6—C14—C19—C18	−176.5 (2)

### Hydrogen-bond geometry (Å, °)

**Table:**

*D*—H···*A*	*D*—H	H···*A*	*D*···*A*	*D*—H···*A*
C10—H10A···O3	0.97	2.26	3.026 (3)	135
C10—H10A···O3^i^	0.97	2.55	3.165 (3)	122
C10—H10B···O3^ii^	0.97	2.44	3.279 (3)	145
C19—H19A···N5^iii^	0.93	2.61	3.491 (3)	158

Symmetry codes: (i) −*x*, −*y*, −*z*+1; (ii) −*x*, *y*+1/2, −*z*+1/2; (iii) *x*, −*y*−1/2, *z*−1/2.
